# Effects of an online mindfulness-based intervention on brain haemodynamics: a pilot randomized controlled trial using functional near-infrared spectroscopy

**DOI:** 10.1093/cercor/bhae321

**Published:** 2024-08-15

**Authors:** Koichiro Adachi, Ryu Takizawa

**Affiliations:** Department of Clinical Psychology, Graduate School of Education, The University of Tokyo, 7-3-1, Hongo, Bunkyo-ku, Tokyo, 113-0033, Japan; Department of Clinical Psychology, Graduate School of Education, The University of Tokyo, 7-3-1, Hongo, Bunkyo-ku, Tokyo, 113-0033, Japan; MRC Social, Genetic and Developmental Psychiatry Centre, Institute of Psychiatry, Psychology and Neuroscience, King’s College London, Strand, London, WC2R 2LS, United Kingdom

**Keywords:** hemodynamic response, meditation, oxygenated hemoglobin, prefrontal cortex, verbal fluency task

## Abstract

Although many neuroimaging studies have evaluated changes in the prefrontal cortex during mindfulness-based interventions, most of these studies were cross-sectional studies of skilled participants or involved pre–post comparisons before and after a single session. While functional near-infrared spectroscopy is a useful tool to capture changes in the hemodynamic response of the prefrontal cortex during continuous mindfulness-based intervention, its ability to detect the accumulated effects of continuous mindfulness-based intervention is currently unclear. We investigated whether a 12-wk online mindfulness-based intervention changed the hemodynamic response of the prefrontal cortex during a verbal fluency task. Eighty-two healthy university students were randomly allocated to a 12-wk online mindfulness-based intervention group or a wait-list control group. The integral values of oxygenated hemoglobin measured using functional near-infrared spectroscopy before and after the intervention were compared to the values in the wait-list group. The intervention condition showed significantly greater functional near-infrared spectroscopy signal activation than the control condition; however, the effect sizes before and after the intervention were small. Thus, continuous mindfulness-based intervention could alter prefrontal cortex function, and functional near-infrared spectroscopy could be useful for measuring the accumulated effects of continuous mindfulness-based interventions. With a better understanding of the association between mindfulness and functional near-infrared spectroscopy signals, functional near-infrared spectroscopy can be used for biofeedback analyses.

## Introduction

Interest in the role of mindfulness in mental health has grown recently. Mindfulness is defined as the awareness that emerges through paying attention on purpose, in the present moment, and non-judgmentally to the unfolding of experience moment by moment (Kabat-Zinn 2003). Mindfulness consists of two components: self-regulation of attention and orientation to experience (Bishop et al. 2004). Self-regulation of attention involves sustained attention, attention switching, and the inhibition of elaborative processing. Orientation to experience involves adopting a particular orientation toward one’s experiences in the present moment and is characterized by curiosity, openness, and acceptance. Mindfulness-based interventions (MBIs) have been shown to be effective for various mental health problems. Previous studies have shown that MBIs can decrease psychological distress, anxiety, and depression and increase quality of life in both clinical (Khoury et al. 2013; Goyal et al. 2014) and nonclinical populations (Khoury et al. 2015; Galante et al. 2021).

Methods to increase the availability of mindfulness-based approaches include the dissemination of brief online MBIs. The application of formal MBIs, such as Mindfulness-based Stress Reduction (MBSR) and Mindfulness-based Cognitive Therapy, to healthy populations is difficult because formal MBIs require sufficient time and commitment. However, online MBIs have recently seen a surge in interest because of their availability. Online MBIs have been shown to have a medium effect on stress and small effects on depression, anxiety, well-being, and mindfulness (Spijkerman et al. 2016).

Mindfulness has also been shown to change psychological and neurophysiological indices. Many studies have reported an association between mindfulness and prefrontal cortex (PFC) functioning (Hölzel et al. 2011; Zeidan et al. 2011). PFC function is closely associated with mental health. The PFC modulates emotion-generative systems such as the amygdala, which is responsible for the detection of affectively arousing stimuli (Ochsner and Gross 2005). Davidson and McEwen (2012) indicated that social stress increases amygdala volume and decreases PFC volume and that cognitive therapy or mindfulness may improve them.

Neuroimaging studies have demonstrated the effects of mindfulness on PFC function (Boccia et al. 2015). Meditation experts have a thicker PFC and right anterior insular cortex, which correlates with their meditation experience (Lazar et al. 2005). Tang et al. (2015) demonstrated that mindfulness meditation is characterized by active cognitive regulation in meditation beginners, who need to overcome habitual ways of reacting internally to their emotions and may, therefore, show greater prefrontal activation. They described that, in the early stages of meditation training, achieving the meditation state seemed to involve the use of attentional control and mental effort; thus, areas of the lateral prefrontal and parietal cortices were more active than before training. Systematic reviews have shown that MBIs can activate the PFC and improve emotion regulation (Fox et al. 2016; Gotink et al. 2016).

In the domain of mindfulness neuroscience research, functional magnetic resonance imaging (fMRI) and electroencephalography (EEG) are primarily used to uncover neural activity in mindfulness. In particular, fMRI has been commonly used in mindfulness neuroscience studies because fMRI can monitor the hemodynamic and metabolic changes associated with neural activity with impressive spatial resolution in a noninvasive manner (Irani et al. 2007) compared to the low spatial resolution of EEG. However, fMRI is physically constraining, susceptible to motion artifacts, exposes participants to loud noises, and is expensive. These characteristics make fMRI unsuitable for certain research applications and untenable for many clinical applications (Irani et al. 2007). Therefore, most fMRI studies evaluating the effects of mindfulness are conducted only once and target meditation experts (e.g. Lazar et al. 2005; Hölzel et al. 2007; Brewer et al. 2011; Hasenkamp and Barsalou 2012). However, such studies are subject to potential confounding factors owing to their cross-sectional designs. Moreover, the definition of meditation experts may encompass a variety of participants because such studies cannot control the content and duration of their meditation. Therefore, these studies could not determine whether the effects detected were due to mindfulness meditation.

Although some recent fMRI studies have measured the effects of multi-session MBIs, most of these studies did not include a control group (e.g. Haase et al. 2015; Tomasino and Fabbro 2016) or had small sample sizes (e.g. Hölzel et al. 2013, *n* = 26; Creswell et al. 2016, *n* = 35) due to the low availability of fMRI. Young et al. (2018), who reviewed studies that measured the effects of an eight-session MBI with fMRI, found that among the seven studies included, three were randomized controlled trials (RCTs), two were controlled trials, and two were “before and after” trials with no control group. In the assessment of sample sizes, the mean sample size per study before and after the MBI was 17.71 (SD = 7.11), and that before and after the control conditions was 15.5 (SD = 5.94). One of the key considerations for neuroimaging studies on MBIs is the inclusion of larger sample sizes and control conditions. Thus, RCTs with sufficient sample sizes are required to improve the quality of evidence.

Functional near-infrared spectroscopy (fNIRS) has attracted attention as an alternative that can address some of the limitations of fMRI and EEG. fNIRS offers a noninvasive and safe method for monitoring brain activity. In addition, fNIRS is more compact, portable, and less expensive than fMRI, and fNIRS has sufficient spatial resonance compared to EEG. Because of its compact and portable design, fNIRS allows for more ecologically valid investigations of brain function in clinical environments (Irani et al. 2007). In addition, fNIRS can be easily used in longitudinal studies owing to its relative insensitivity to movement artifacts, easy applicability, and high versatility compared to fMRI. Such studies are important for examining treatment effects following pharmacological, psychotherapeutic, and neurophysiological interventions (Ehlis et al. 2014). Using optical sensors, fNIRS can detect hemodynamic changes in the PFC. Activation of the lateral PFC (LPFC) can especially serve as an index for successful psychotherapy because LPFC activity is known to increase with the employment of executive functions, such as working memory, inhibition, and emotional control (Ozawa 2021).

Although fNIRS is a good method for measuring the effects of MBIs, empirical research on fNIRS measurements of hemodynamics during mindfulness practice is limited (Choo et al. 2019). Furthermore, most of these studies used fNIRS in a single session. Deepeshwar et al. (2014) conducted an fNIRS study in 22 healthy male volunteers and showed an increase in oxy-hemoglobin (oxy-Hb) and total hemoglobin (total-Hb) concentrations with reduced deoxy-hemoglobin (deoxy-Hb) concentration over the right PFC during meditation. Miyashiro et al. (2021) conducted a pre–post comparison study on 17 healthy volunteers and showed that meditation activated the orbitofrontal cortex.

Although these pre–post comparisons of the findings before and after single sessions can capture state changes, these changes may be transient and do not clarify whether repeated state changes induced by multi-session interventions could lead to the trait changes that are usually considered the outcome of psychotherapy (Ozawa 2021). Ozawa (2021) asserted that measurements taken in multiple sessions should address this issue. Gagrani et al. (2018) conducted an RCT in patients with primary open-angle glaucoma and reported that fNIRS showed a significant improvement in oxy-Hb changes in the PFC of the intervention group than the control group. However, no study has used fNIRS to show that multi-session MBI can change hemodynamics in healthy populations.

MBI studies using fNIRS need to employ an RCT design and include a sufficient sample size to provide more rigorous empirical evidence. Choo et al. (2019) highlighted that more rigorous research and evaluation are needed to establish the use of fNIRS to examine brain hemodynamics as objective evidence. RCTs are the gold standard in clinical research and are commonly used to validate empirical evidence regarding the effectiveness of psychotherapy. In addition, almost all MBI studies using fNIRS are limited by their small sample sizes, which limits the ability to draw definitive conclusions regarding the relationship between MBI and fNIRS signals (e.g. *n* = 22 in Deepeshwar et al. 2014 and *n* = 17 in Miyashiro et al. 2021).

Thus, this study aimed to use fNIRS to determine whether sustained mindfulness practice could improve the hemodynamics of the PFC in an RCT.

## Materials and methods

### Study design

This study was designed as a crossover-design RCT with a wait-list control group. Undergraduate and graduate students at Japanese universities were recruited through open calls on university websites. Eligible participants were allocated to either a 12-wk self-help and online MBI group or a wait-list control group. The data were collected at the laboratory between June 2021 and January 2022. The study protocol was approved by the Ethics Committee of the University of Tokyo (No. 21-18, Dated 2021 May 11).

### Participants

The criteria for inclusion were as follows: (1) undergraduate or graduate students in Japanese universities, (2) aged ≥18 yr, (3) not currently diagnosed with a mental disorder, (4) K6 (Japanese version) score <13 (Kessler et al. 2002; Furukawa et al. 2008) (to exclude participants with clinically severe depressive/anxiety symptoms), and (5) ability to participate in measurements at the laboratory.

A total of 100 participants applied for this study. Six participants dropped out before random allocation because they had K6 scores ≥13. A total of 94 participants were included in this study. Among these 94 participants, 47 each were randomly assigned to the intervention and control groups. Each participant completed the pre-test questionnaires via a web form before undergoing measurements at the laboratory. The fNIRS measurements were performed after the participants provided written informed consent. Participants in the intervention group underwent breathing meditation once at the laboratory (T0). We excluded the T0 data of participants who dropped out because they were out of contact before the T0 measurement (intervention group, *n* = 4; control group, *n* = 5), experienced technical issues with fNIRS (intervention group, *n* = 2), or withdrew informed consent later (intervention group, *n* = 1). Thus, the T0 data of 82 participants (intervention group, *n* = 40; control group, *n* = 42) were included in this study.

Participants in the intervention group began their daily mindfulness practice and continued it for 12 wk. The participants in the control group were asked to spend the control period as usual. After 12 wk, the participants completed the post-test questionnaire and fNIRS measurements. Participants in the control group were trained in breathing meditation once in the laboratory (T1). We excluded the T1 data of participants who experienced technical issues with fNIRS (intervention group, *n* = 2; control group, *n* = 2) and those who did not complete the post-intervention questionnaire (intervention group, *n* = 11; control group, *n* = 3). Thus, the data of 65 participants were included in the T1 dataset (intervention group, *n* = 27; control group, *n* = 38).

After the control period, participants in the control group completed the post-test questionnaire and fNIRS measurements and then started their daily mindfulness practice for 12 wk. After 12 wk, the participants in the control group completed a post-intervention questionnaire and underwent fNIRS measurements (T2). We excluded the T2 data of participants who experienced technical issues with fNIRS (*n* = 2) and those who did not complete the post-intervention questionnaire (*n* = 5). Thus, the post-intervention data of 31 participants at T2 were included in the analysis. A total of 96 pre–post data points were included in the condition comparison analysis (intervention condition, *n* = 58; control condition, *n* = 38).

### Online MBI

The MBI was conducted using audio on a website. We sent identifiers (IDs) and passwords to each participant. Participants entered their IDs and passwords on the website and listened to the audio recordings daily. Participants were taught how to practice online and underwent breathing meditation once while listening to the audio at the laboratory before the intervention period. The content was changed every month (every 4 wk). The participants listened to “Breathing Meditation” (5 min 31 s) for the first month, “Body Scan Meditation” (3 min 34 s) for the next month, and “Body and Sound Meditation” (3 min 47 s) for the last month. Each meditation session was ~5 min long. These pre-recorded audio mindfulness meditations were created from Japanese scripts based on those used by the UCLA Mindful Awareness Research Center (2021). The number of days each participant logged in was counted as their adherence to practice. As the intervention was designed as an “intention to treat” trial, adherence to daily practice was not mandatory over the entire period.

### Activation task

The verbal fluency task (VFT) was used to accurately evaluate the effects of MBIs using fNIRS. The VFT is a cognitive task used as a neuropsychological test or neuroimaging task. The VFT has been shown to significantly increase oxy-Hb levels in certain PFC areas over both hemispheres in comparison with baseline resting conditions in healthy individuals (Herrmann et al. 2003, 2006). The VFT is the most popular paradigm in psychiatric settings (Dieler et al. 2012). Patients with major depressive disorder show significantly less oxy-Hb activation during the VFT than healthy controls (Suto et al. 2004; Zhang et al. 2015). Patients with other psychiatric disorders, such as schizophrenia, bipolar disorder, autism spectrum disorder, and attention-deficit/hyperactivity disorder, also show activation characteristics different from those of healthy controls (Kameyama et al. 2006; Kuwabara et al. 2006; Marumo et al. 2014; Takizawa et al. 2014; Husain et al. 2023). Oxy-Hb activation has been shown to be negatively correlated with depression severity (Noda et al. 2012) and positively correlated with social functioning (Takizawa et al. 2008; Kinou et al. 2013) in patients with psychiatric disorders. In nonclinical populations, oxy-Hb activation has been suggested to have a significant negative correlation with subjective depression and a significant positive correlation with quality of life (Sawa et al. 2013; Satomura et al. 2014).

**Fig. 1:**
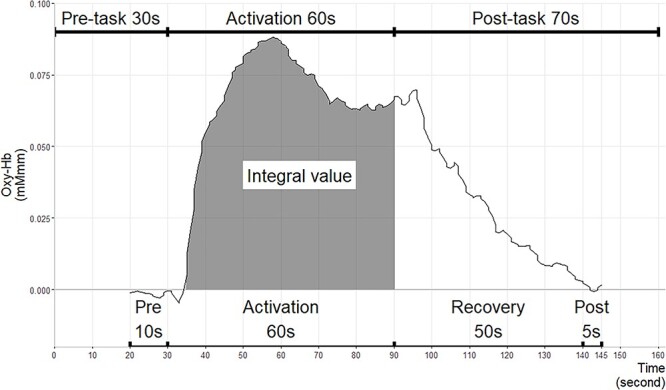
Waveform, integral value, task period, and analysis period.

The activation task during fNIRS measurement in this study was similar to that used in previous studies (Takizawa et al. 2008; Takizawa et al. 2014). For the letter version of the VFT, we used a block design with three 160-s blocks, each consisting of a 30-s pre-task period, a 60-s activation period, and a 70-s post-task period. The analysis included a 10-s pre-task period, a 60-s activation period, and a 30-s post-task period (Fig. 1).

Each participant was seated in a chair in front of a monitor for all of the measurements. Participants were asked to avoid body movements or knitting their brows during the fNIRS measurements because of the potential for artifacts unrelated to the study task. The participants were then told to relax with their eyes open and fixate on a monitor in front of them. In the letter version of the VFT, the participant was instructed to retrieve and speak out as loud as many words as possible, beginning with the Japanese syllabic characters (hiragana) on the monitor, in a 60-s activation period. The characters were /Ha/, /A/, and /To/ at T0; /O/, /Ki/, and /Se/ at T1; and /Ta/, /Ha/, and /O/ at T2. The three characters were changed every 20 s during the activation period. The total number of words was counted as the VFT performance. In the pre- and post-task period, the participant was asked to repeat simple character strings (/a/, /i/, /u/, /e/, and /o/).

### Measurements

#### Sociodemographic variables

Participants’ age, sex, dominant hand, grade, marriage, living status (alone or with others), household income, psychiatric history, disease, medicines, and previous relaxation practice experience, including mindfulness, were recorded using a questionnaire. The number of years of education of the participants’ parents was also obtained as an indicator of socioeconomic status.

#### Functional near-infrared spectroscopy

**Fig. 2:**
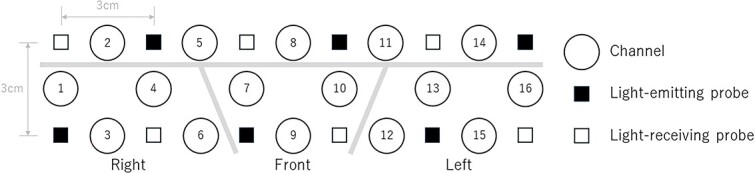
Functional near-infrared spectroscopy settings.

Sixteen-channel fNIRS (OEG-16; Spectratech Inc., Yokohama, Japan) measurements were obtained to record changes in oxy-Hb, deoxy-Hb, and total-Hb levels. fNIRS uses two wavelengths (~770 and 840 nm) of near-infrared light. Absorption was recorded to estimate the oxy-Hb levels. The temporal resolution was set at 650 ms. This system could measure changes in hemodynamics 3 cm below the scalp. Six light-emitting and six light-receiving probes were arranged in a 6 (width) × 2 (length) matrix on the participants’ foreheads (Fig. 2). The distance between the light-emitting and light-receiving probes was 3.0 cm. The center of the probe matrix was placed at Fpz (the midpoint between Fp1 and Fp2) in accordance with the international 10–20 system for recording electroencephalograms. The measurement ranges included Fp1, Fpz, Fp2, F7, F8, and the lower regions of F3 and F4: Fp1 and Fp2 corresponded to the left and right superior or middle frontal gyri (BA10), F7 and F8 to the inferior frontal gyrus (BA47), and F3 and F4 to the superior or middle frontal gyrus (BA9 and 10) (Okamoto et al. 2004). After the probes were mounted, calibration was performed several times, and channels that remained outside the specified values or were unused were excluded.

The 11 middle and lower channels (Ch1, 3, 4, 6, 7, 9, 10, 12, 13, 15, and 16) were used for the analysis because the upper-channel data (Ch2, 5, 8, 11, and 14), located in a hair-covered area, were not obtained with a sufficient signal-to-noise ratio owing to the lack of near-infrared light. In this study, we analyzed the fNIRS data for the following three regions of interest (ROIs): Ch1, 3, 4, and 6 for the right channel (right PFC); Ch7, 9, and 10 for the middle channel (middle PFC); and Ch12, 13, 15, and 16 for the left channel (left PFC). The average value of the target channels in the ROI was considered as the fNIRS signal change in the ROI. Studies of fNIRS measurements during the VFT have demonstrated that group and cluster analyses have sufficient test–retest reliability, whereas retest reliability is not satisfactory at the single-subject and single-channel levels (Schecklmann et al. 2008).

The modified Beer–Lambert Law was used to convert optical densities into changes in oxy-Hb and deoxy-Hb concentrations. Oxy-Hb measurements were used for the analysis since they are the most sensitive measures of changes in regional cerebral blood flow (Hoshi et al. 2001), are highly correlated with changes in fMRI measurements during cognitive tasks, and are thought to reflect brain activity (Strangman et al. 2002; Cui et al. 2011). Evidence also shows that retest reliability is the highest for oxy-Hb measurements (Plichta et al. 2006).

The BRainAnalyzer (B.R. Systems Inc., Kamakura, Japan) was used to analyze the fNIRS data. The raw oxy-Hb data were bandpass filtered. The high-pass filter was set to 0.008 Hz, and the low-pass filter was set to 0.2 Hz. For artifact rejection, all differences between the sampling data and their standard deviations were calculated. Trials that included at least one difference value over ±5 SDs were excluded from the statistical analysis (Ozawa et al. 2014), with 73.37% of the total trials remaining. VFT waveforms were calculated with a 10-s pre-task period, a 60-s activation-task period, a 50-s recovery period, and a 5-s post-task period (Fig. 1). Baseline linear fitting was applied to the data between the pre- and post-task periods. Integral values were calculated as indicators of changes in oxy-Hb levels using parametric statistical tests. The integral value describes the size of the hemodynamic response during the 60-s activation-task period (Takizawa et al. 2014). After the artifact channels were removed, the average value was calculated for each ROI with one or more channels remaining in the ROI.

### Analytical methods

An RCT was conducted using a crossover design. First, the mean and SD of the integral values of each ROI under the intervention and control conditions were calculated. Second, baseline sociodemographic variables, VFT performance scores, and integral values were descriptively analyzed (means and SDs). Baseline differences across the groups were examined using *t*-tests for continuous variables. For categorical values, when at least one cell had expected values of <5, the baseline differences across groups were examined using Fisher’s exact test. Baseline differences across the groups were examined using the chi-square test. Third, the changes before and after the intervention or wait-list control were estimated using paired *t*-tests. Cohen’s *d* effect size was used, with cutoff values of 0.20, 0.50, and.80 for small, medium, and large effect sizes, respectively (Cohen 1988). To compare the changes in integral values across conditions, we used multiple-regression analysis after controlling for the baseline integral values. The model regressed fNIRS outcomes on intervention group assignment (0 for mindfulness training and 1 for wait-list control), considering the baseline integral value as a covariate. In comparisons showing any significant baseline differences, we additionally controlled for baseline variables that were significantly different between groups to verify the robustness of the results. All tests were two tailed with an alpha level of 0.05. Statistical analyses were conducted using R Studio version 2023.6.0.421 (Posit team 2023) and R version 4.2.2 (R Core Team 2022).

## Results

### Demographic characteristics and baseline values

Table 1 shows the baseline demographic and clinical characteristics of the participants who completed the post-test measurements (T2) in this study. The sample included 65 participants (mean age, 21.82 yr [SD, 4.31 yr]; females, 46.2%; living alone, 43%; right dominant hand, 86%). Eight participants had previous experience in some form of relaxation methods. Eight participants reported a psychiatric history for themselves and/or their family members (three for themselves and six for their family members). Only one participant was married. A *t*-test showed the absence of significant age differences between the groups (*P* = 0.16), and a chi-square test showed the absence of significant sex-related differences between the groups (*P* = 0.60). In addition, no other demographic characteristics showed significant differences between the groups. The two groups showed no significant differences in task performance or baseline integral values. On average, participants in the intervention condition practiced for a total of 24.03 d [SD, 26.72 d].

**Table 1:** Demographic and clinical characteristics and baseline values.

	Total		Intervention group		Control group		Group difference			
	Mean	SD	Mean	SD	Mean	SD	Test	Estimate	*df*	*P*-value
*N*	65		27		38					
Age	21.82	4.31	22.70	6.16	21.18	2.12	*t*	1.41	63	0.16
Sex, female/male	30/35		14/13		16/22		*χ^2^*	0.27	1	0.60
Dominant hand, right/left	56/9		25/2		31/7		*Fisher*	2.78		0.29
Course, undergraduate/graduate	49/16		22/5		27/11		*χ^2^*	0.45	1	0.50
Living, alone/with others	28/37		9/18		19/19		*χ^2^*	1.17	1	0.28
Psychiatric history, yes/no	57/8		23/4		34/4		*Fisher*	0.68		0.71
Relaxation experience, yes/no	8/57		4/23		4/34		*Fisher*	0.68		0.71
Parent education, yr	16.02	1.69	16.04	1.68	16.00	1.72	*t*	0.09	63	0.93
Household income, million yen	8.67	3.44	9.00	3.42	8.43	3.48	*t*	0.64	61	0.52
Task performance	16.41	4.49	16.63	5.00	16.25	4.13	*t*	0.33	61	0.74
Integral values (front PFC)	2.82	2.81	2.61	2.49	2.97	3.04	*t*	−0.47	53	0.64
Integral values (left PFC)	3.26	2.83	3.15	2.52	3.34	3.07	*t*	−0.26	57	0.80
Integral values (right PFC)	3.34	3.00	3.02	2.53	3.57	3.32	*t*	−0.67	53	0.50

*Abbreviations*: PFC, prefrontal cortex

### Test of the differences between the intervention and control conditions

**Fig. 3:**
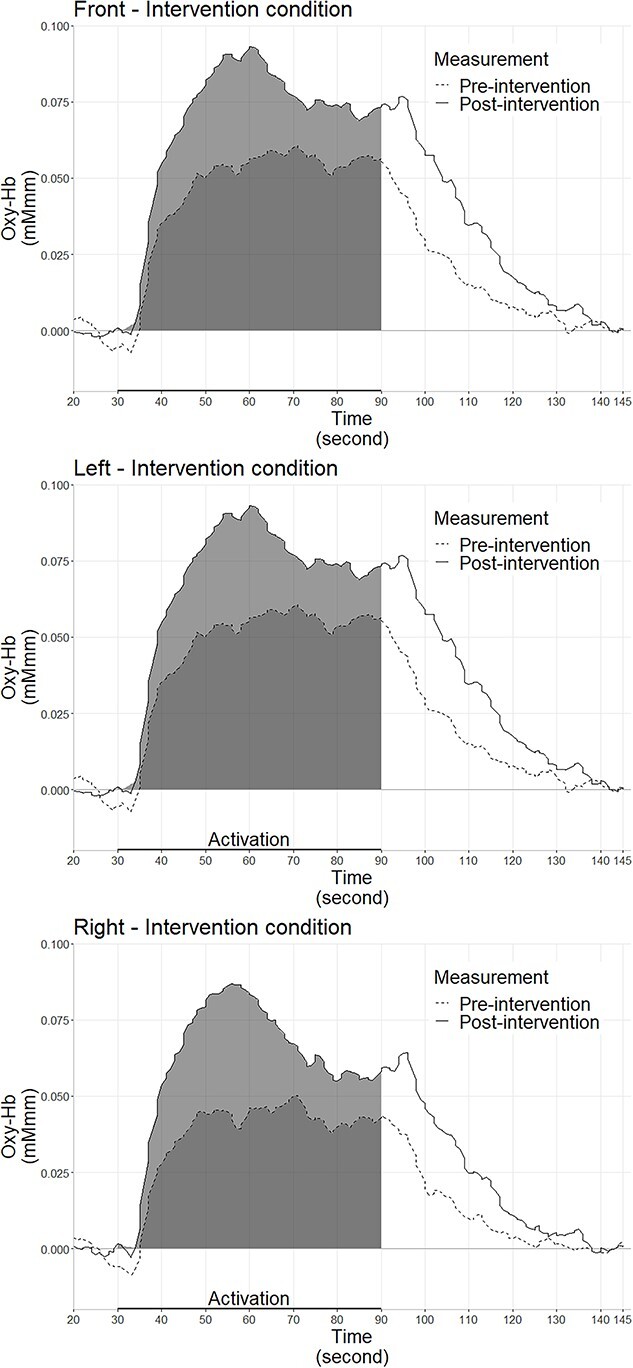
The average hemodynamic waveforms of all participants before and after the intervention for each region of interest (ROI). The integral values are the areas shown in gray.

Table 2 shows the mean and SD values of the integral values, pre–post comparisons using paired *t*-tests in the intervention condition, and between-group comparisons using multiple-regression analysis. The oxy-Hb waveforms in each ROI are shown in Fig. 3. After artifact rejection, data from 58 participants in the intervention condition (front PFC, *n* = 47; left PFC, *n* = 51; right PFC, *n* = 50) and 38 participants in the control condition (front PFC, *n* = 29; left PFC, *n* = 33; right PFC, *n* = 31) were included in the analysis.

**Table 2:** Comparison of long-term changes in integral values between the intervention and control conditions.

	Intervention condition (*n* = 58)				Pre/post comparison				Control condition (*n* = 38)				Group comparison			
	Pre		Post		*t*-value	*df*	*P*-value	Cohen’s *d*	Pre		Post		*b*	SE	*t*-value	*P*-value
Front PFC	2.29	3.22	3.71	4.30	2.21	46	0.03^*^	0.37	3.04	2.52	2.11	3.57	−1.98	0.89	−2.22	0.03^*^
Left PFC	2.89	2.84	3.80	3.88	1.76	50	0.08^†^	0.26	3.39	3.10	2.68	3.01	−1.40	0.71	−1.97	0.05^†^
Right PFC	2.44	2.88	3.57	4.01	2.10	49	0.04^*^	0.32	3.50	3.36	2.17	3.10	−1.98	0.77	−2.58	0.01^*^

*Abbreviations*: PFC, prefrontal cortex

^†^ *P* < 0.10; ^*^P < 0.05; ^**^P < 0.01; ^***^P < 0.001

Paired *t*-tests revealed that participants in the intervention condition demonstrated significant pre–post-intervention increases in the integral values of the front PFC (*t*(46) = 2.21, *P* = 0.03, Cohen’s *d* = 0.37) and right PFC (*t*(49) = 2.10, *P* = 0.05, Cohen’s *d* = 0.32) and marginally significant increases in the left PFC (*t*(50) = 1.76, *P* = 0.08, Cohen’s *d* = 0.26). All effect sizes were small.

**Fig. 4:**
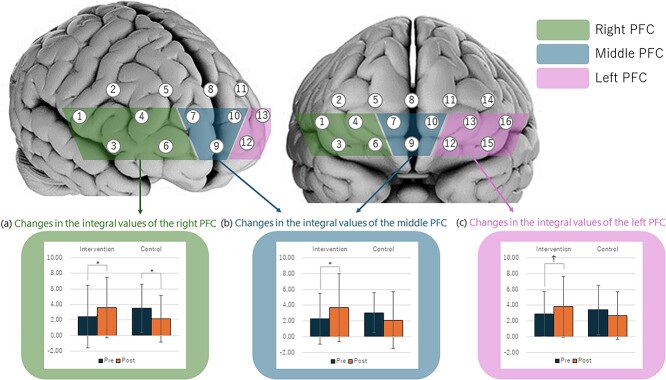
The measurement positions used in the present study and the changes in the integral values of each ROI. The channel numbers are indicated above the corresponding measurement locations. The ROIs of the prefrontal cortex (PFC) are also noted. (a) Changes in the integral values of the left PFC. (b) Changes in the integral values of the middle PFC. (c) Changes in the integral values of the right PFC. ^†^*P* < 0.10; ^*^P < 0.05.

Participants in the intervention condition demonstrated a significant increase in the integral values of the front PFC (*b* = −1.98, *t*(73) = −2.22, *P* = 0.03) and right PFC (*b* = −1.98, *t*(78) = −2.58, *P* = 0.01) in comparison with the control condition after controlling for the pre-test integral values. The participants in the intervention condition demonstrated a marginally significant increase in the left PFC (*b* = −1.40, *t*(81) = −1.97, *P* = 0.05) in comparison with those in the control condition after controlling for the pre-test integral values. These changes in the integral values of each ROI are shown in Fig. 4.

## Discussion

This is the first fNIRS study to detect long-term hemodynamic changes by multi-session mindfulness practices in a healthy population. These RCT results generally showed that participants in the intervention condition demonstrated a significant fNIRS signal increase in the PFC in comparison with the wait-list control condition during the VFT. These findings suggest that continuous mindfulness practice as a daily routine may increase the hemodynamic response in the PFC, and this increase may be detectable using fNIRS.

The finding that MBI can increase PFC activation is consistent with the results of previous fMRI reviews (Fox et al. 2016; Young et al. 2018) and an fNIRS review (Choo et al. 2019). However, most studies have only demonstrated that meditation experts activate their PFC more than non-practitioners or that one-session mindfulness practice can increase the amount of blood flow in the PFC. Thus far, research on the accumulated effects of continuous mindfulness practice is limited. Young et al. (2018) reviewed multi-session MBI studies using fMRI and showed that multi-session MBIs could activate the PFC. However, their recommendations were limited by the absence of RCTs or the small sample sizes in the studies included in the review. These limitations may be attributable to the low availability of fMRI. Moreover, no rigorous empirical studies have examined the accumulated effects of continuous mindfulness practice in healthy populations, despite the high availability of fNIRS. This is the first study to provide evidence of the effect of multi-session MBI on PFC hemodynamics in healthy populations.

In this study, the effect sizes for all ROIs were small, and the ROIs showed almost no differences. Although no quantitative meta-analysis has been conducted in multi-session MBI studies due to the wide variety of outcome measures and participants (Gotink et al. 2016), the small effect sizes were consistent with results obtained in a few previous studies. Goldin et al. (2021) investigated the effects of MBSR on fMRI in patients with social anxiety disorder. Eight-week MBSR was associated with a significant increase in BOLD percentage signal change in PFC regions when reappraising (partial *η*^2^ = 0.01; small effect) and accepting (partial *η*^2^ = 0.08; medium effect) negative self-beliefs. Although direct comparisons cannot be made due to the different nature of the tasks, they showed roughly similar effect sizes. Young et al. (2018) also documented a lack of consistent evidence for the particular areas or networks involved. However, our results showed that the effects on the middle and right ROIs were slightly greater than those on the left ROI. The finding that MBI may increase the amount of blood flow in the right PFC is consistent with those of previous fMRI studies (e.g. Hasenkamp and Barsalou 2012; Haase et al. 2015; Tomasino and Fabbro 2016) and EEG studies (e.g. Faber et al. 2015). Deepeshwar et al. (2014) also used fNIRS to show an increase in the oxy-Hb concentration over the right PFC during a single session of meditation.

This finding might indicate effects specific to meditation. A previous study showed that brief relaxation practices can activate the right and left frontopolar PFC and the left orbitofrontal cortex during arithmetic tasks, although it was a single session (Zhang et al. 2020). The right PFC might be activated mainly by mindfulness practices, and the left PFC might be activated mainly by simple relaxation practices. Neuroimaging studies have suggested that tasks of sustained attention and vigilance are initiated via activity in the PFC, particularly in the right hemisphere, as well as in the anterior cingulate cortex (Pardo et al. 1991; Petersen and Posner 2012). Since meditation requires an intense focus of attention, meditation has been proposed to begin with the activation of the PFC, particularly in the right hemisphere (Newberg and Iversen 2003). Though the elements of meditation consist of focused attention (FA) and open monitoring (OM), FA meditation has been suggested to be associated with increased activity in the right PFC (Lutz et al. 2008; Hasenkamp and Barsalou 2012; Lippelt et al. 2014). FA meditation aims to direct and sustain attention to a selected object, while OM meditation is aimed at nonreactive metacognitive monitoring (Lutz et al. 2008). In this study, “Breathing Meditation” and “Body Scan Meditation” were closer to FA meditation, and “Body and Sound Meditation” included part of FA meditation. Thus, the results of this study suggest that FA meditation could activate the PFC, particularly in the right hemisphere, and that this activation could be detected by fNIRS.

Another possible explanation is that activation was attenuated in the left PFC through repetition of the VFT. As described by Young et al. (2018), evidence for the involvement of specific areas is lacking, and some studies have shown that multi-session MBI could also increase the amount of blood flow in the left PFC (Gagrani et al. 2018; Zheng et al. 2019). Kawakubo et al. (2018) demonstrated that repetition of the VFT significantly attenuated task-induced activation in the left PFC. They suggested that the attenuation was due to improved neural efficiency in manipulating the VFT because the left PFC is a central region for verbal processing.

### Strengths

A large number of published studies (e.g. Hölzel et al. 2011; Zeidan et al. 2011) have described the effects of mindfulness practices on PFC function. These effects were mainly measured using fMRI. However, fMRI lacks ecological validity because of large physical constraints and susceptibility to artifacts. Therefore, while such studies have shown that one-session mindfulness practice affects PFC function, rigorous empirical studies on the effects of multi-session mindfulness practice have been lacking. fNIRS can measure PFC activity in a simpler manner while retaining higher ecological validity and sufficient spatial resolution. Interest in fNIRS has increased in the past 20 yr, mainly in the field of psychiatry. Although fNIRS is a potentially useful tool for measuring PFC activity, few empirical studies have measured the effects of mindfulness practice using fNIRS (Choo et al. 2019). Gagrani et al. (2018) conducted an RCT to measure the effects of 6-wk face-to-face mindfulness practice in glaucoma patients and showed that fNIRS could detect significant oxy-Hb changes in the mindfulness group in comparison with the control group. However, no previous study could clarify how fNIRS could be used to detect the effects of mindfulness practices in healthy individuals. This study demonstrated for the first time that fNIRS can detect significant oxy-Hb changes as the accumulated effect of continuing a 12-wk online mindfulness practice program in healthy individuals. The key strengths of the present study were the scientific rigor based on its RCT methodology and the larger sample size.

### Limitations

However, this study also had several limitations. First, the spatial resolution of fNIRS was insufficient to accurately annotate the channel locations in the PFC region. Because the PFC is the main target of fNIRS measurements, this technique cannot measure the effects on other brain structures that are deeply associated with mindfulness. Thus, the relationship with brain function, as suggested in this study, needs to be examined in more detail. Second, fNIRS signals are expected to include motion artifacts and scalp vascular artifacts, which are mainly influenced by the autonomic nervous system (e.g. heart rate, respiration, and blood pressure) (Kirilina et al. 2012; Haeussinger et al. 2014). Third, the completion rate was low because of dropout and technical issues associated with the use of fNIRS. There was also low adherence to the online MBI. These low completion rates and low adherence may have been sources of bias. Fourth, our population consisted predominantly of urban Japanese university students, and this study targeted a healthy population. Therefore, the applicability of our findings to diverse populations, such as older adults and those with mental health disorders, remains unclear. The generalizability of our findings to other populations and ethnic groups should be examined in future studies. Fifth, this study employed a crossover RCT design to enhance the statistical power. In a crossover design, the intervention group was treated as the control group after the intervention and washout periods. However, because studies that verify the effects of psychological interventions generally cannot remove the effects of interventions, this study employed a one-way crossover design similar to most psychological intervention studies. The analysis of the parallel design showed no significant group differences but in the same direction as that of the crossover design.

### Future perspectives

These findings highlight the potential usefulness of fNIRS in monitoring the accumulated effects of multi-session MBIs. A recent meta-analysis reported that over half of the studies found no significant effects of MBIs on self-reported mindfulness scales from pre- to post-intervention (Visted et al. 2015). In addition, studies on MBIs in active control conditions have shown no significant advantage in increasing self-reported mindfulness. Nevertheless, self-reported measures are prone to recall bias (Stone and Shiffman 2002) and social desirability bias (Paulhus 2017). In addition, questionnaire measures that request individuals to provide accounts of their own experiences depend on their practice and experience in interrogating their minds, which is viewed as a skill that changes with the MBI (Davidson and Kaszniak 2015). While self-reported measures have had some success as a method of measuring the effects of MBIs, supplementary objective measures are also valuable for measuring the effects of MBIs more accurately.

Although fMRI can be used to measure the effects of MBIs, its limited availability limits its use in real-world clinical practice. In contrast, the measurement of PFC hemodynamics using fNIRS is a more portable, less time-consuming, and less invasive approach than approaches based on other neurophysiological indices. Therefore, fNIRS may be used as a monitoring biomarker, i.e. “a biomarker measured serially to assess the status of a disease or medical condition or for evidence of exposure to (or effect of) a medical product or an environmental agent” (FDA-NIH Biomarker Working Group. 2016). By allowing for repeated measurements, fNIRS can monitor responses to MBIs more easily as a biomarker. In the present study, we tried to capture the continuous effects of mindfulness meditation in an RCT by simple measurements in the frontal cortex. Based on our findings, further research should explore the underlying mechanism of how brain hemodynamics is altered by continuous mindfulness meditation. Connectivity analysis with fMRI or whole-head fNIRS with more channels is a promising line of research. In addition, interest in resting-state cerebral hemodynamic fluctuations or connectivity has increased in recent studies. Several studies have demonstrated that mindfulness meditation alters the resting-state functional connectivity of brain networks implicated in mind wandering (the default mode network) and executive control (the executive control network) (e.g. Creswell et al. 2016). Most of the studies used fMRI to measure resting-state functional connectivity. However, previous research has confirmed the feasibility of using functional connectivity with whole-head fNIRS during the resting state (Mesquita et al. 2010). It would be interesting to examine the effects of continuous MBI during the resting-state task using whole-head fNIRS.

Furthermore, with a greater understanding of the neurophysiological mechanisms underlying continuous mindfulness practice, fNIRS measurements have the potential to be used as biofeedback to monitor daily mindfulness practice and enhance psychotherapy with a combination of both biofeedback and mindfulness (Choo et al. 2019). fNIRS can be used to investigate hemodynamic activity in the brain by detecting changes in oxy-Hb and deoxy-Hb concentrations. Hemodynamic activity reflects the brain’s neuronal signal with a delay of a few seconds, a mechanism known as neurovascular coupling. Increased activity in specific brain areas increases blood supply and oxy-Hb levels (Irani et al. 2007). It has been observed that fNIRS signals reach 50% of the maximum level 1 to 4 s later than DC-magnetoencephalography signals, which can detect the brain’s neuronal signal (Mackert et al. 2008). Therefore, real-time biofeedback is possible with fNIRS and is being attempted. A pilot study showed activation in the left and central parts of the PFC when comparing mindfulness tasks and baseline tasks. The findings were consistent with prevailing fMRI studies, and the authors concluded that fNIRS is promising for studying real-time neurophysiological cortical activations in MBIs (Yu et al. 2020). When a combination of biofeedback and mindfulness is developed, audio feedback may be considered rather than feedback from a visual display because mindfulness meditation is generally practiced with eyes closed.

In conclusion, the present study demonstrated that a 12-wk online MBI changed hemodynamic responses in the PFC, as detected by fNIRS. This rigorous empirical study suggested that continuous mindfulness practices may have accumulative effects on PFC function in healthy populations. It also showed that fNIRS could characterize the hemodynamic effect of continuous mindfulness practice, indicating the potential of fNIRS for biofeedback assessments. Further studies should explore the associations between changes in fNIRS signals and subjective or other objective indicators. These findings have implications for measuring the effectiveness of MBIs in clinical settings.

## References

[ref1] Bishop SR , Lau M, Shapiro S, Carlson L, Anderson ND, Carmody J, Segal ZV, Abbey S, Speca M, Velting D, et al. Mindfulness: a proposed operational definition. Clin Psychol Sci Pract. 2004:11(3):230–241. 10.1093/clipsy.bph077.

[ref2] Boccia M , Piccardi L, Guariglia P. The meditative mind: a comprehensive meta-analysis of MRI studies. Bio Med Res Int. 2015:2015:1–11. 10.1155/2015/419808. PMC4471247 26146618

[ref3] Brewer JA , Worhunsky PD, Gray JR, Tang Y-Y, Weber J, Kober H. Meditation experience is associated with differences in default mode network activity and connectivity. Proc Natl Acad Sci USA. 2011:108(50):20254–20259. 10.1073/pnas.1112029108. 22114193 PMC3250176

[ref4] Choo CC , Lee JJW, Kuek JHL, Ang KK, Yu JH, Ho CS, Ho RC. Mindfulness and hemodynamics in Asians: a literature review. Asian J Psychiatr. 2019:44:112–118. 10.1016/j.ajp.2019.07.035. 31369945

[ref5] Cohen J . Statistical power analysis for the behavioral sciences. 2nd ed. Hillsdale, N.J: Lawrence Erlbaum Associates; 1988.

[ref6] Creswell JD , Taren AA, Lindsay EK, Greco CM, Gianaros PJ, Fairgrieve A, Marsland AL, Brown KW, Way BM, Rosen RK, et al. Alterations in resting-state functional connectivity link mindfulness meditation with reduced interleukin-6: a randomized controlled trial. Biol Psychiatry. 2016:80(1):53–61. 10.1016/j.biopsych.2016.01.008. 27021514

[ref7] Cui X , Bray S, Bryant DM, Glover GH, Reiss AL. A quantitative comparison of NIRS and fMRI across multiple cognitive tasks. NeuroImage. 2011:54(4):2808–2821. 10.1016/j.neuroimage.2010.10.069. 21047559 PMC3021967

[ref8] Davidson RJ , Kaszniak AW. Conceptual and methodological issues in research on mindfulness and meditation. Am Psychol. 2015:70(7):581–592. 10.1037/a0039512. 26436310 PMC4627495

[ref9] Davidson RJ , McEwen BS. Social influences on neuroplasticity: stress and interventions to promote well-being. Nat Neurosci. 2012:15(5):689–695. 10.1038/nn.3093. 22534579 PMC3491815

[ref10] Deepeshwar S , Vinchurkar SA, Visweswaraiah NK, Nagendra HR. Hemodynamic responses on prefrontal cortex related to meditation and attentional task. Front Syst Neurosci. 2014:8:252. 25741245 10.3389/fnsys.2014.00252 PMC4330717

[ref11] Dieler AC , Tupak SV, Fallgatter AJ. Functional near-infrared spectroscopy for the assessment of speech related tasks. Brain Lang. 2012:121(2):90–109. 10.1016/j.bandl.2011.03.005. 21507475

[ref12] Ehlis A-C , Schneider S, Dresler T, Fallgatter AJ. Application of functional near-infrared spectroscopy in psychiatry. NeuroImage. 2014:85(Pt 1):478–488. 10.1016/j.neuroimage.2013.03.067. 23578578

[ref13] Faber PL , Lehmann D, Gianotti LRR, Milz P, Pascual-Marqui RD, Held M, Kochi K. Zazen meditation and no-task resting EEG compared with LORETA intracortical source localization. Cogn Process. 2015:16(1):87–96. 10.1007/s10339-014-0637-x. 25284209

[ref14] FDA-NIH Biomarker Working Group . BEST (biomarkers, EndpointS, and other tools) resource [internet]. Silver Spring (MD): Food and Drug Administration (US). [accessed 2024 Apr 12]. https://www.ncbi.nlm.nih.gov/books/NBK326791/. 2016. 27010052

[ref15] Fox KCR , Dixon ML, Nijeboer S, Girn M, Floman JL, Lifshitz M, Ellamil M, Sedlmeier P, Christoff K. Functional neuroanatomy of meditation: a review and meta-analysis of 78 functional neuroimaging investigations. Neurosci Biobehav Rev. 2016:65:208–228. 10.1016/j.neubiorev.2016.03.021. 27032724

[ref16] Furukawa TA , Kawakami N, Saitoh M, Ono Y, Nakane Y, Nakamura Y, Tachimori H, Iwata N, Uda H, Nakane H, et al. The performance of the Japanese version of the K6 and K10 in the world mental health survey Japan. Int J Methods Psychiatr Res. 2008:17(3):152–158. 10.1002/mpr.257. 18763695 PMC6878390

[ref17] Gagrani M , Faiq MA, Sidhu T, Dada R, Yadav RK, Sihota R, Kochhar KP, Verma R, Dada T. Meditation enhances brain oxygenation, upregulates BDNF and improves quality of life in patients with primary open angle glaucoma: a randomized controlled trial. Restor Neurol Neurosci. 2018:36(6):741–753. 10.3233/RNN-180857. 30400122

[ref18] Galante J , Friedrich C, Dawson AF, Modrego-Alarcón M, Gebbing P, Delgado-Suárez I, Gupta R, Dean L, Dalgleish T, White IR, et al. Mindfulness-based programmes for mental health promotion in adults in nonclinical settings: a systematic review and meta-analysis of randomised controlled trials. PLoS Med. 2021:18(1):e1003481. 10.1371/journal.pmed.1003481. 33428616 PMC7799763

[ref19] Goldin PR , Thurston M, Allende S, Moodie C, Dixon ML, Heimberg RG, Gross JJ. Evaluation of cognitive behavioral therapy vs mindfulness meditation in brain changes during reappraisal and acceptance among patients with social anxiety disorder: a randomized clinical trial. JAMA Psychiatry. 2021:78(10):1134–1142. 10.1001/jamapsychiatry.2021.1862. 34287622 PMC8295897

[ref20] Gotink RA , Meijboom R, Vernooij MW, Smits M, Hunink MGM. 8-Week mindfulness based stress reduction induces brain changes similar to traditional long-term meditation practice - a systematic review. Brain Cogn. 2016:108:32–41. 10.1016/j.bandc.2016.07.001. 27429096

[ref21] Goyal M , Singh S, Sibinga EMS, Gould NF, Rowland-Seymour A, Sharma R, Berger Z, Sleicher D, Maron DD, Shihab HM, et al. Meditation programs for psychological stress and well-being: a systematic review and meta-analysis. JAMA Intern Med. 2014:174(3):357–368. 10.1001/jamainternmed.2013.13018. 24395196 PMC4142584

[ref22] Haase L , May AC, Falahpour M, Isakovic S, Simmons AN, Hickman SD, Liu TT, Paulus MP. A pilot study investigating changes in neural processing after mindfulness training in elite athletes. Front Behav Neurosci. 2015:9:229. 10.3389/fnbeh.2015.00229. 26379521 PMC4550788

[ref23] Haeussinger FB , Dresler T, Heinzel S, Schecklmann M, Fallgatter AJ, Ehlis A-C. Reconstructing functional near-infrared spectroscopy (fNIRS) signals impaired by extra-cranial confounds: an easy-to-use filter method. NeuroImage. 2014:95:69–79. 10.1016/j.neuroimage.2014.02.035. 24657779

[ref24] Hasenkamp W , Barsalou LW. Effects of meditation experience on functional connectivity of distributed brain networks. Front Hum Neurosci. 2012:6:38. 10.3389/fnhum.2012.00038. 22403536 PMC3290768

[ref25] Herrmann MJ , Ehlis A-C, Fallgatter AJ. Frontal activation during a verbal-fluency task as measured by near-infrared spectroscopy. Brain Res Bull. 2003:61(1):51–56. 10.1016/S0361-9230(03)00066-2. 12788206

[ref26] Herrmann MJ , Walter A, Ehlis A-C, Fallgatter AJ. Cerebral oxygenation changes in the prefrontal cortex: effects of age and gender. Neurobiol Aging. 2006:27(6):888–894. 10.1016/j.neurobiolaging.2005.04.013. 16023767

[ref27] Hölzel BK , Ott U, Hempel H, Hackl A, Wolf K, Stark R, Vaitl D. Differential engagement of anterior cingulate and adjacent medial frontal cortex in adept meditators and non-meditators. Neurosci Lett. 2007:421(1):16–21. 10.1016/j.neulet.2007.04.074. 17548160

[ref28] Hölzel BK , Lazar SW, Gard T, Schuman-Olivier Z, Vago DR, Ott U. How does mindfulness meditation work? Proposing mechanisms of action from a conceptual and neural perspective. Perspect Psychol Sci. 2011:6(6):537–559. 10.1177/1745691611419671. 26168376

[ref29] Hölzel BK , Hoge EA, Greve DN, Gard T, Creswell JD, Brown KW, Barrett LF, Schwartz C, Vaitl D, Lazar SW. Neural mechanisms of symptom improvements in generalized anxiety disorder following mindfulness training. NeuroImage Clin. 2013:2:448–458. 10.1016/j.nicl.2013.03.011. 24179799 PMC3777795

[ref30] Hoshi Y , Kobayashi N, Tamura M. Interpretation of near-infrared spectroscopy signals: a study with a newly developed perfused rat brain model. J Appl Physiol. 2001:90(5):1657–1662. 10.1152/jappl.2001.90.5.1657. 11299252

[ref31] Husain SF , Chiang SK, Vasu AA, Goh CP, McIntyre RS, Tang TB, Tran BX, Dang THT, Nguyen TT, Ho RC, et al. Functional near-infrared spectroscopy of English-speaking adults with attention-deficit/hyperactivity disorder during a verbal fluency task. J Atten Disord. 2023:27(13):1448–1459. 10.1177/10870547231180111. 37341192

[ref32] Irani F , Platek SM, Bunce S, Ruocco AC, Chute D. Functional near infrared spectroscopy (fNIRS): an emerging neuroimaging technology with important applications for the study of brain disorders. Clin Neuropsychol. 2007:21(1):9–37. 10.1080/13854040600910018. 17366276

[ref33] Kabat-Zinn J . Mindfulness-based interventions in context: past, present, and future. Clin Psychol Sci Pract. 2003:10(2):144–156. 10.1093/clipsy.bpg016.

[ref34] Kameyama M , Fukuda M, Yamagishi Y, Sato T, Uehara T, Ito M, Suto T, Mikuni M. Frontal lobe function in bipolar disorder: a multichannel near-infrared spectroscopy study. NeuroImage. 2006:29(1):172–184. 10.1016/j.neuroimage.2005.07.025. 16125979

[ref35] Kawakubo Y , Yanagi M, Tsujii N, Shirakawa O. Repetition of verbal fluency task attenuates the hemodynamic activation in the left prefrontal cortex: enhancing the clinical usefulness of near-infrared spectroscopy. PLoS One. 2018:13(3):e0193994. 10.1371/journal.pone.0193994. 29561889 PMC5862477

[ref36] Kessler RC , Andrews G, Colpe LJ, Hiripi E, Mroczek DK, Normand SLT, Walters EE, Zaslavsky AM. Short screening scales to monitor population prevalences and trends in non-specific psychological distress. Psychol Med. 2002:32(6):959–976. 10.1017/S0033291702006074. 12214795

[ref37] Khoury B , Lecomte T, Fortin G, Masse M, Therien P, Bouchard V, Chapleau M-A, Paquin K, Hofmann SG. Mindfulness-based therapy: a comprehensive meta-analysis. Clin Psychol Rev. 2013:33(6):763–771. 10.1016/j.cpr.2013.05.005. 23796855

[ref38] Khoury B , Sharma M, Rush SE, Fournier C. Mindfulness-based stress reduction for healthy individuals: a meta-analysis. J Psychosom Res. 2015:78(6):519–528. 10.1016/j.jpsychores.2015.03.009. 25818837

[ref39] Kinou M , Takizawa R, Marumo K, Kawasaki S, Kawakubo Y, Fukuda M, Kasai K. Differential spatiotemporal characteristics of the prefrontal hemodynamic response and their association with functional impairment in schizophrenia and major depression. Schizophr Res. 2013:150(2–3):459–467. 10.1016/j.schres.2013.08.026. 24016725

[ref40] Kirilina E , Jelzow A, Heine A, Niessing M, Wabnitz H, Brühl R, Ittermann B, Jacobs AM, Tachtsidis I. The physiological origin of task-evoked systemic artefacts in functional near infrared spectroscopy. NeuroImage. 2012:61(1):70–81. 10.1016/j.neuroimage.2012.02.074. 22426347 PMC3348501

[ref41] Kuwabara H , Kasai K, Takizawa R, Kawakubo Y, Yamasue H, Rogers MA, Ishijima M, Watanabe K, Kato N. Decreased prefrontal activation during letter fluency task in adults with pervasive developmental disorders: a near-infrared spectroscopy study. Behav Brain Res. 2006:172(2):272–277. 10.1016/j.bbr.2006.05.020. 16806521

[ref42] Lazar SW , Kerr CE, Wasserman RH, Gray JR, Greve DN, Treadway MT, McGarvey M, Quinn BT, Dusek JA, Benson H, et al. Meditation experience is associated with increased cortical thickness. Neuroreport. 2005:16(17):1893–1897. 10.1097/01.wnr.0000186598.66243.19. 16272874 PMC1361002

[ref43] Lippelt DP , Hommel B, Colzato LS. Focused attention, open monitoring and loving kindness meditation: effects on attention, conflict monitoring, and creativity - a review. Front Psychol. 2014:5:1083. 10.3389/fpsyg.2014.01083. 25295025 PMC4171985

[ref44] Lutz A , Slagter HA, Dunne JD, Davidson RJ. Attention regulation and monitoring in meditation. Trends Cogn Sci. 2008:12(4):163–169. 10.1016/j.tics.2008.01.005. 18329323 PMC2693206

[ref45] Mackert B , Leistner S, Sander T, Liebert A, Wabnitz H, Burghoff M, Trahms L, Macdonald R, Curio G. Dynamics of cortical neurovascular coupling analyzed by simultaneous DC-magnetoencephalography and time-resolved near-infrared spectroscopy. NeuroImage. 2008:39(3):979–986. 10.1016/j.neuroimage.2007.09.037. 17997330

[ref46] Marumo K , Takizawa R, Kinou M, Kawasaki S, Kawakubo Y, Fukuda M, Kasai K. Functional abnormalities in the left ventrolateral prefrontal cortex during a semantic fluency task, and their association with thought disorder in patients with schizophrenia. NeuroImage. 2014:85(Pt 1):518–526. 10.1016/j.neuroimage.2013.04.050. 23624170

[ref47] Mesquita RC , Franceschini MA, Boas DA. Resting state functional connectivity of the whole head with near-infrared spectroscopy. Biomed Opt Express. 2010:1(1):324–336. 10.1364/BOE.1.000324. 21258470 PMC3005169

[ref48] Miyashiro S , Yamada Y, Muta T, Ishikawa H, Abe T, Hori M, Oka K, Koshikawa F, Ito E. Activation of the orbitofrontal cortex by both meditation and exercise: a near-infrared spectroscopy study. PLoS One. 2021:16(2):e0247685. 10.1371/journal.pone.0247685. 33621250 PMC7901739

[ref49] Newberg AB , Iversen J. The neural basis of the complex mental task of meditation: neurotransmitter and neurochemical considerations. Med Hypotheses. 2003:61(2):282–291. 10.1016/S0306-9877(03)00175-0. 12888320

[ref50] Noda T , Yoshida S, Matsuda T, Okamoto N, Sakamoto K, Koseki S, Numachi Y, Matsushima E, Kunugi H, Higuchi T. Frontal and right temporal activations correlate negatively with depression severity during verbal fluency task: a multi-channel near-infrared spectroscopy study. J Psychiatr Res. 2012:46(7):905–912. 10.1016/j.jpsychires.2012.04.001. 22572569

[ref51] Ochsner KN , Gross JJ. The cognitive control of emotion. Trends Cogn Sci. 2005:9(5):242–249. 10.1016/j.tics.2005.03.010. 15866151

[ref52] Okamoto M , Dan H, Sakamoto K, Takeo K, Shimizu K, Kohno S, Oda I, Isobe S, Suzuki T, Kohyama K, et al. Three-dimensional probabilistic anatomical cranio-cerebral correlation via the international 10-20 system oriented for transcranial functional brain mapping. NeuroImage. 2004:21(1):99–111. 10.1016/j.neuroimage.2003.08.026. 14741647

[ref53] Ozawa S . Application of near-infrared spectroscopy for evidence-based psychotherapy. Front Psychol. 2021:12:527335. 10.3389/fpsyg.2021.527335. 34366946 PMC8342759

[ref54] Ozawa S , Matsuda G, Hiraki K. Negative emotion modulates prefrontal cortex activity during a working memory task: a NIRS study. Front Hum Neurosci. 2014:8:46. 10.3389/fnhum.2014.00046. 24574991 PMC3918646

[ref55] Pardo JV , Fox PT, Raichle ME. Localization of a human system for sustained attention by positron emission tomography. Nature. 1991:349(6304):61–64. 10.1038/349061a0. 1985266

[ref56] Paulhus DL . Socially desirable responding on self-reports. In: Zeigler-Hill V, Shackelford TK, editors. Encyclopedia of personality and individual differences. Cham: Springer International Publishing; 2017. p. 1–5, 10.1007/978-3-319-28099-8_1349-1.

[ref57] Petersen SE , Posner MI. The attention system of the human brain: 20 years after. Annu Rev Neurosci. 2012:35(1):73–89. 10.1146/annurev-neuro-062111-150525. 22524787 PMC3413263

[ref58] Plichta MM , Herrmann MJ, Baehne CG, Ehlis A-C, Richter MM, Pauli P, Fallgatter AJ. Event-related functional near-infrared spectroscopy (fNIRS): are the measurements reliable? NeuroImage. 2006:31(1):116–124. 10.1016/j.neuroimage.2005.12.008. 16446104

[ref59] Posit team . Rstudio: integrated development environment for R. 2023. [accessed 2024 Apr 12]. http://www.posit.co/.

[ref60] R Core Team . R: a language and environment for statistical computing. 2022. [accessed 2024 Apr 12]. https://www.R-project.org/.

[ref61] Satomura Y , Takizawa R, Koike S, Kawasaki S, Kinoshita A, Sakakibara E, Nishimura Y, Kasai K. Potential biomarker of subjective quality of life: prefrontal activation measurement by near-infrared spectroscopy. Soc Neurosci. 2014:9(1):63–73. 10.1080/17470919.2013.861359. 24294926

[ref62] Sawa M , Yamashita H, Fujimaki K, Okada G, Takahashi T, Yamawaki S. Negative correlation between affective symptoms and prefrontal activation during a verbal fluency task: a near-infrared spectroscopy study. Neuropsychobiology. 2013:67(2):103–110. 10.1159/000345161. 23407267

[ref63] Schecklmann M , Ehlis A-C, Plichta MM, Fallgatter AJ. Functional near-infrared spectroscopy: a long-term reliable tool for measuring brain activity during verbal fluency. NeuroImage. 2008:43(1):147–155. 10.1016/j.neuroimage.2008.06.032. 18657624

[ref64] Spijkerman MPJ , Pots WTM, Bohlmeijer ET. Effectiveness of online mindfulness-based interventions in improving mental health: a review and meta-analysis of randomised controlled trials. Clin Psychol Rev. 2016:45:102–114. 10.1016/j.cpr.2016.03.009. 27111302

[ref65] Stone AA , Shiffman S. Capturing momentary, self-report data: a proposal for reporting guidelines. Ann Behav Med. 2002:24(3):236–243. 10.1207/S15324796ABM2403_09. 12173681

[ref66] Strangman G , Culver JP, Thompson JH, Boas DA. A quantitative comparison of simultaneous BOLD fMRI and NIRS recordings during functional brain activation. NeuroImage. 2002:17(2):719–731. 10.1006/nimg.2002.1227. 12377147

[ref67] Suto T , Fukuda M, Ito M, Uehara T, Mikuni M. Multichannel near-infrared spectroscopy in depression and schizophrenia: cognitive brain activation study. Biol Psychiatry. 2004:55(5):501–511. 10.1016/j.biopsych.2003.09.008. 15023578

[ref68] Takizawa R , Kasai K, Kawakubo Y, Marumo K, Kawasaki S, Yamasue H, Fukuda M. Reduced frontopolar activation during verbal fluency task in schizophrenia: a multi-channel near-infrared spectroscopy study. Schizophr Res. 2008:99(1–3):250–262. 10.1016/j.schres.2007.10.025. 18063344

[ref69] Takizawa R , Fukuda M, Kawasaki S, Kasai K, Mimura M, Pu S, Noda T, Niwa S-I, Okazaki Y, Joint Project for Psychiatric Application of Near-Infrared Spectroscopy (JPSY-NIRS) Group. Joint project for psychiatric application of near-infrared spectroscopy (JPSY-NIRS) group. Neuroimaging-aided differential diagnosis of the depressive state. NeuroImage. 2014:85(Pt 1):498–507. 10.1016/j.neuroimage.2013.05.126. 23764293

[ref70] Tang Y-Y , Hölzel BK, Posner MI. The neuroscience of mindfulness meditation. Nat Rev Neurosci. 2015:16(4):213–225. 10.1038/nrn3916. 25783612

[ref71] Tomasino B , Fabbro F. Increases in the right dorsolateral prefrontal cortex and decreases the rostral prefrontal cortex activation after-8 weeks of focused attention based mindfulness meditation. Brain Cogn. 2016:102:46–54. 10.1016/j.bandc.2015.12.004. 26720411

[ref72] UCLA Mindful Awareness Research Center . Free guided meditations. 2021. [accessed 2024 Mar 30]. https://www.uclahealth.org/programs/marc/free-guided-meditations/guided-meditations.

[ref73] Visted E , Vøllestad J, Nielsen MB, Nielsen GH. The impact of group-based mindfulness training on self-reported mindfulness: a systematic review and meta-analysis. Mindfulness. 2015:6(3):501–522. 10.1007/s12671-014-0283-5.

[ref74] Young KS , van der Velden AM, Craske MG, Pallesen KJ, Fjorback L, Roepstorff A, Parsons CE. The impact of mindfulness-based interventions on brain activity: a systematic review of functional magnetic resonance imaging studies. Neurosci Biobehav Rev. 2018:84:424–433. 10.1016/j.neubiorev.2017.08.003. 28797556

[ref75] Yu J , Ang KK, Choo CC, Ho CS, Ho R, So RQ. Prefrontal cortical activation while doing mindfulness task: a pilot functional near-infrared spectroscopy study. Annu Int Conf IEEE Eng Med Biol Soc. 2020:2020:2905–2908. 10.1109/EMBC44109.2020.9175464. 33018614

[ref76] Zeidan F , Martucci KT, Kraft RA, Gordon NS, McHaffie JG, Coghill RC. Brain mechanisms supporting the modulation of pain by mindfulness meditation. J Neurosci. 2011:31(14):5540–5548. 10.1523/JNEUROSCI.5791-10.2011. 21471390 PMC3090218

[ref77] Zhang H , Dong W, Dang W, Quan W, Tian J, Chen R, Zhan S, Yu X. Near-infrared spectroscopy for examination of prefrontal activation during cognitive tasks in patients with major depressive disorder: a meta-analysis of observational studies. Psychiatry Clin Neurosci. 2015:69(1):22–33. 10.1111/pcn.12209. 24897940

[ref78] Zhang Z , Olszewska-Guizzo A, Husain SF, Bose J, Choi J, Tan W, Wang J, Xuan Tran B, Wang B, Jin Y, et al. Brief relaxation practice induces significantly more prefrontal cortex activation during arithmetic tasks comparing to viewing greenery images as revealed by functional near-infrared spectroscopy (fNIRS). Int J Environ Res Public Health. 2020:17(22):8366. 10.3390/ijerph17228366. PMC7698004 33198147

[ref79] Zheng Y-L , Wang D-X, Zhang Y-R, Tang Y-Y. Enhancing attention by synchronizing respiration and fingertip pressure: a pilot study using functional near-infrared spectroscopy. Front Neurosci. 2019:13:1209. 10.3389/fnins.2019.01209. 31780888 PMC6861189

